# Transcriptional changes induced by bevacizumab combination therapy in responding and non-responding recurrent glioblastoma patients

**DOI:** 10.1186/s12885-017-3251-3

**Published:** 2017-04-18

**Authors:** Thomas Urup, Line Mærsk Staunstrup, Signe Regner Michaelsen, Kristoffer Vitting-Seerup, Marc Bennedbæk, Anders Toft, Lars Rønn Olsen, Lars Jønson, Shohreh Issazadeh-Navikas, Helle Broholm, Petra Hamerlik, Hans Skovgaard Poulsen, Ulrik Lassen

**Affiliations:** 1 Department of Radiation Biology, The Finsen Center, Section 6321, Rigshospitalet, Blegdamsvej 9, DK-2100 Copenhagen, Denmark; 2 Section for Computational and RNA biology (SCARB), Department of Biology, University of Copenhagen, Ole Maaløesvej 5, DK-2200 Copenhagen, Denmark; 3 Center for Genomic Medicine, Rigshospitalet, Blegdamsvej 9, DK-2100 Copenhagen, Denmark; 4 Department of Biology, The Bioinformatics Centre, University of Copenhagen, Ole Maaløesvej 5, DK-2200 Copenhagen, Denmark; 5 Department of Systems Biology, Center for Biological Sequence Analysis, Technical University of Denmark, Kemitorvet, Building 208, DK-2800 Lyngby, Denmark; 6 Neuroinflammation Unit, BRIC, University of Copenhagen, Ole Maaløesvej 5, DK-2100 Copenhagen, Denmark; 7 Department of Pathology, Center of Diagnostic Investigation, Rigshospitalet, Blegdamsvej 9, DK-2100 Copenhagen, Denmark; 8 Brain Tumor Biology Group, Danish Cancer Society Research Center, Strandboulevarden 49, DK-2100 Copenhagen, Denmark; 9 Department of Oncology, The Finsen Center, Rigshospitalet, Blegdamsvej 9, DK-2100 Copenhagen, Denmark; 10 Phase I Unit, The Finsen Center, Rigshospitalet, Blegdamsvej 9, DK-2100 Copenhagen, Denmark

**Keywords:** Anti-angiogenic, RNA-sequencing, Protein kinase C, Reverse mesenchymal transition, TGF-beta

## Abstract

**Background:**

Bevacizumab combined with chemotherapy produces clinical durable response in 25–30% of recurrent glioblastoma patients. This group of patients has shown improved survival and quality of life. The aim of this study was to investigate changes in gene expression associated with response and resistance to bevacizumab combination therapy.

**Methods:**

Recurrent glioblastoma patients who had biomarker-accessible tumor tissue surgically removed both before bevacizumab treatment and at time of progression were included. Patients were grouped into responders (*n* = 7) and non-responders (*n* = 14). Gene expression profiling of formalin-fixed paraffin-embedded tumor tissue was performed using RNA-sequencing.

**Results:**

By comparing pretreatment samples of responders with those of non-responders no significant difference was observed. In a paired comparison analysis of pre- and posttreatment samples of non-responders 1 gene was significantly differentially expressed. In responders, this approach revealed 256 significantly differentially expressed genes (72 down- and 184 up-regulated genes at the time of progression). Genes differentially expressed in responders revealed a shift towards a more proneural and less mesenchymal phenotype at the time of progression.

**Conclusions:**

Bevacizumab combination treatment demonstrated a significant impact on the transcriptional changes in responders; but only minimal changes in non-responders. This suggests that non-responding glioblastomas progress chaotically without following distinct gene expression changes while responding tumors adaptively respond or progress by means of the same transcriptional changes. In conclusion, we hypothesize that the identified gene expression changes of responding tumors are associated to bevacizumab response or resistance mechanisms.

**Electronic supplementary material:**

The online version of this article (doi:10.1186/s12885-017-3251-3) contains supplementary material, which is available to authorized users.

## Background

Glioblastoma is the most malignant primary brain tumor in adults. Standard treatment comprises surgery followed by radio-chemotherapy with concomitant and adjuvant temozolomide. Despite this aggressive treatment the prognosis is dismal with a median survival of 14.6 months [1]. Recurrence of glioblastoma is almost inevitable and at recurrence numerous agents have shown limited clinical effect [2].

Glioblastoma is characterized by excessive and aberrant angiogenesis. Anti-angiogenic agents inhibiting vascular endothelial growth factor A (VEGF) have been shown to normalize the tumor vasculature and improve blood flow and drug delivery [3, 4]. This emphasizes the potential value of combining anti-angiogenic therapy with drugs targeting the tumor. Bevacizumab, a VEGF targeting antibody, in combination with chemotherapy is among the most frequently used treatments in recurrent glioblastoma patients. Although this treatment regimen has not proved active in the total population of recurrent glioblastoma patients [5], 25–30% of the patients achieve treatment response (defined as radiological and clinical improvement). This group of patients has demonstrated improved survival as well as quality of life [6–9], highlighting the importance of identifying predictive biomarkers for bevacizumab efficacy.

Glioblastoma consists of a mixture of cancer cell subclones, glial cells, stromal cells and immune cells, and each of these cell populations adds to the tumor heterogeneity. This complicates the interpretation of tumor biomarker analysis. Nevertheless, gene expression profiling of glioblastoma has identified four molecular subtypes, namely Neural, Proneural, Classical and Mesenchymal, and preliminary evidence indicates survival benefit in distinct molecular subtypes treated with bevacizumab combination therapy [10, 11]. However, the results of these two studies have been inconsistent and we have along with others shown that the subtypes do not impact bevacizumab response in recurrent glioblastoma [12, 13].

Due to the rarity of paired, biomarker evaluable, recurrent glioblastoma tissue samples, our current knowledge on bevacizumab response and resistance mechanisms is based on preclinical animal studies and small clinical case reports [14–19]. Recently, novel gene expression technologies, including RNA-sequencing (RNA-Seq), have shown high performance on formalin-fixed paraffin embedded (FFPE) glioblastoma samples [10, 11]. This will prove valuable for future clinical biomarker studies on archived tumor tissue.

In this study, we hypothesized that bevacizumab combination treatment exerts selective pressure on the tumors and creates adaptive transcriptional changes as tumors respond and progress. Accordingly, the aim was to identify transcriptional changes by RNA-Seq in paired tumor samples, before and after bevacizumab treatment in both responding and non-responding recurrent glioblastoma patients.

## Methods

### Patients

All patients with glioblastoma (pathologically confirmed WHO grade IV) treated at recurrence with bevacizumab plus irinotecan at Rigshospitalet in the period between May 2005 and December 2014, were assessed for eligibility. Eligibility criteria for this study were 1) response evaluability and 2) biomarker accessible tumor tissue prior to bevacizumab treatment and at time of progression after bevacizumab treatment. The criteria are specified below.

### Treatment and clinical follow-up

Treatment at recurrence followed Danish national guidelines and was planned at a multidisciplinary team conference. If the neurosurgeons considered the tumor amenable for relapse surgery, this was offered in order to remove as much tumor tissue as possible. Bevacizumab and irinotecan therapy was administered according to a published treatment protocol [20]. Prior to initiation of treatment the patients had to have measurable progressive disease by contrast-enhanced MRI after standard therapy and had to be at least 4 weeks from prior chemotherapy and 3 months from completion of radiation therapy. Clinical and radiological follow-up was performed according to protocol [20]. Treatment response was evaluated based on the RANO criteria and response was confirmed on the subsequent follow-up MRI [21]. Responders were defined as patients with complete or partial response (CR + PR) and non-responders were defined as patients with stable disease (SD) or progressive disease (PD).

### Sample acquisition and RNA preparation

A total of 264 patients were assessed for eligibility. Twenty-four response-evaluable patients had surgery before and after bevacizumab treatment and had archived paired FFPE tissue blocks at the Department of Pathology, Rigshospitalet. Tissue review was conducted by a neuropathologist, who was blinded to clinical outcome. The number of tumor cells was estimated based on hematoxylin and eosin-staining. Macrodissection was performed in a few cases to remove large amounts of normal brain tissue and only samples containing a tumor cell frequency > 50% were selected for RNA-extraction. If tumor blocks from relapse surgery prior to bevacizumab treatment were available and contained sufficient amount of tumor cells, they were included in preference to tumor blocks from time of glioblastoma diagnosis. All post-bevacizumab samples were obtained from relapse surgery following progression on bevacizumab treatment and no intermediate relapse therapy was administered. Three patients had an insufficient number of tumor cells in one of the paired tumor blocks and were excluded prior to analysis. Thus, a total of 21 patients with paired tumor blocks were included in the study. Samples were sectioned into 3 × 10 μm thick FFPE sections and RNA was extracted from paired tumor blocks in three equal sample-sized batches using Deparaffinization Solution (Qiagen, Ca. No. 19093) and RNeasy FFPE kit (Qiagen, Ca. No. 73504). RNA extracts were stored at −80 °C.

### Library preparation for RNA-sequencing

Library preparation was carried out using the strand-specific Ovation Human FFPE RNA-Seq Library Systems from Nugen according to the instructions from the manufacturer. 250 ng of total RNA was used as input material for the cDNA synthesis and the double stranded cDNA was fragmented on the Covaris S2 (Covaris, Inc.) in microtubes using the following settings: duty cycle–10%/Intensity–5/cycles/burst-200 for a total of 180 s. RNA-Seq was performed on Illumina HiSeq 2500 (Illumina) as paired end sequencing 2 × 101 bases in Rapid Mode with 5 samples per run resulting in approximately 175 million paired-end reads per run. Raw data was converted to fastq files using CASAVA v1.8.2.

### Filtering and differential expression analysis

**Fig. 1:**
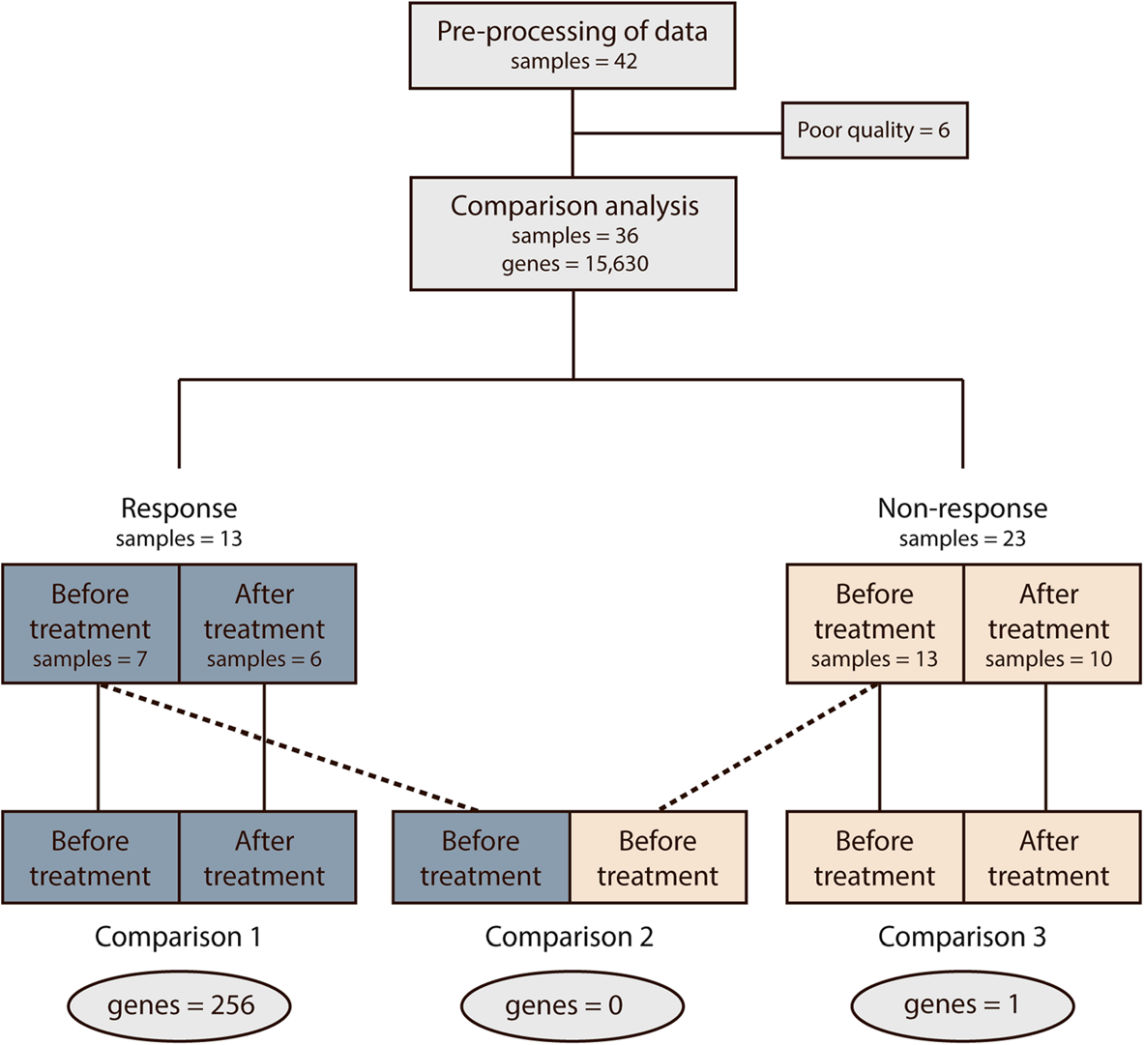
Transcriptional comparison analysis. Flowchart for transcriptional comparison analysis. The number of significantly differentially expressed genes identified is shown below the three analyses

Detailed description of the RNA-Seq analysis can be found in Additional file 1: Supplementary Methods. Briefly, the raw sequencing data was trimmed with Trimmomatic and mapped to the human genome (*hg19*) with TopHat2 [22, 23]. Genes were annotated (Ensemble annotation, release 66) and expression levels were quantified using featureCounts [24]. Libraries with less than 10% of genes having more than 15 fragments were discarded (*n* = 6). Only genes with at least 10 fragments and an abundance of at least 3 Fragments Per Kilobase per Million reads (FPKM) in at least 5 libraries in any of the 4 patient-groups (Fig. 1) were kept for further analysis (15,630 genes). Differential expression analysis was performed using edgeR (v. 3.12.0) either as paired analysis (comparison 1 and 3) or a batch-corrected analysis (comparison 2, see Fig. 1). *P*-values were corrected for multiple testing using the False Discovery Rate (FDR) approach and genes with adjusted *P*-values <0.05 were considered significant. Analysis was performed using the software R version 3.2.2 (R Development Core Team, Vienna, Aurstria, http://www.R-project.org). Expression data are available at the NCBI Geo datasets, accession number GSE79671.

### Gene set enrichment analysis

Gene ontology gene-sets were downloaded (6th Jan 2016) from The European Bioinformatics Institute’s official Gene Ontology mirror. Gene ontology terms from the 5th level of the hierarchical gene ontology term tree were used. Gene sets c2, c3, c6 and H were downloaded from The Molecular Signatures Database (MSigDB [25], via http://bioinf.wehi.edu.au/software/MSigDB/). The enrichment analysis was done using a Fisher’s exact test only considering the 15,630 tested genes, and *P*-values were FDR corrected and adjusted *P*-value <0.05 were considered significant.

### Ingenuity pathway analysis

Differentially expressed genes were analyzed by QIAGEN’s Ingenuity Pathway Analysis (IPA) using the core analysis with default settings and 15,630 tested genes from the RNA-Seq dataset as background (IPA®,QIAGEN Redwood City, www.qiagen.com/ingenuity). The software uses a large database of curated data and computes a score for each network according to the fit of the set of genes supplied in the analysis. The scores were calculated by right-tailed Fisher’s exact test. The scores derived from *P*-values, indicate the likelihood of supplied genes belonging to a network versus those obtained by chance. A consistency score (Z-score) > 2 or < −2 indicates with ≥99% confidence that a supplied gene network was not generated by chance alone. Enrichment of “canonical pathways” and “up-stream regulators” with a Z-score > 2 or < −2 were considered for analysis [26].

## Results

### Patient characteristics

Patient characteristics and clinical outcome are shown in Table 1. All patients had received standard treatment with radio- and chemotherapy (temozolomide) prior to bevacizumab combination therapy. All patients had undergone relapse surgery prior to bevacizumab combination therapy but only half of the resected tumor samples were eligible for biomarker analysis. Consequently, the remaining half of the samples obtained before bevacizumab therapy was from surgery before standard treatment. Patient and sample characteristics did not differ significantly between responders and non-responders. Responders had a significantly longer progression-free survival compared to non-responders (*P* = 0.02) while no significant difference was observed in overall survival (*P* = 0.16).

**Table 1:** Patient characteristics

	All patients (*n =* 21)	Responders (*n =* 7)	Non-responders (*n =* 14)	*P-* value
Gender, *n* (%)				
Male	11 (52)	5 (71)	6 (43)	0.36
Female	10 (48)	2 (29)	8 (57)	
Age, years (range)				
Median	52 (21–70)	53 (35–65)	48 (21–70)	0.66
WHO performance status, *n* (%)				
0	10 (48)	2 (29)	8 (57)	0.42
1	9 (43)	4 (57)	5 (36)	
2	2 (9)	1 (14)	1 (7)	
Secondary glioblastoma, *n* (%)				
Yes	1 (5)	0	1 (7)	1.00
No	20 (95)	7 (100)	13 (93)	
Standard glioblastoma therapy, *n* (%)				
Yes	20 (95)	7 (100)	13 (93)	1.00
No	1 (5)	0	1 (7)	
Prior lines of chemotherapy, *n* (%)				
1	18 (86)	7 (100)	11 (79)	0.52
2	3 (14)	0	3 (21)	
Tumor size, cm^2^ (range)				
Median	9 (1–28)	11 (4–28)	8 (1–16)	0.65
Multifocal disease, *n* (%)				
Yes	2 (10)	0	2 (14)	0.53
No	19 (91)	7 (100)	12 (86)	
Corticosteroid use, *n* (%)^a^				
Yes	14 (67)	3 (43)	11 (79)	0.16
No	7 (33)	4 (57)	3 (21)	
Neurocognitive deficit, *n* (%)				
Yes	8 (38)	3 (43)	4 (29)	0.35
No	13 (62)	4 (57)	10 (71)	
Primary sample, before bevacizumab, *n* (%)				
Initial glioblastoma diagnosis	10 (48)	3 (43)	7 (50)	1.00
Relapse surgery prior to bevacizumab	11 (52)	4 (57)	7 (50)	
Time duration from relapse surgery (after bevacizumab), months				
to initiation of standard therapy, median	17	17	17	0.76
to last bevacizumab administration, median	2	2	2	0.26
Number of bevacizumab treatment cycles^b^				
Median	6	8	6	0.08
Bevacizumab combination therapy, *n* (%)				
Irinotecan	17 (81)	6 (86)	11 (79)	1.00
Irinotecan and cetuximab	4 (19)	1 (14)	3 (21)	
Response, *n* (%)				
Response (CR + PR)	7 (33)	7 (100)	0	
Stable disease	10 (48)	0	10 (71)	
Progressive disease	4 (19)	0	4 (29)	
Progression-free survival, months				
Median	5.4	10.8	3.9	0.02
Overall survival, months				
Median	10.8	14.3	8.6	0.16

*Abbreviations*: *CR* complete response, *PR* partial response

^a^ Prednisolone >10 mg

^b^ Two bevacizumab combination treatments (28 days) defined one treatment cycle

Of the 42 samples, high quality RNA-Seq data was obtained on a total of 36 samples, leaving 20 “pre-bevacizumab samples” and 16 “post-bevacizumab samples” and 16 paired samples. Of the paired samples, 6 patients were classified as responders and 10 patients were classified as non-responders.

### Group comparisons of gene expression profiles

To identify significantly differentially expressed genes between groups, comparison analyses were performed according to a pre-specified analytical strategy, shown in Fig. 1. In the analyses, no confounding effects of clinical factors and no genetic subgroups were identified.

The comparison of pretreatment samples between responders (*n* = 7) and non-responders (*n* = 13) demonstrated no significantly differentially expressed genes.

To identify transcriptional changes at the time of progression compared to before treatment a paired analysis was performed in non-responders (*n* = 10) and responders (*n* = 6), separately: In non-responders, 1 gene was significantly upregulated at the time of progression (Additional file 2: Table S1). In responders, a total of 256 genes were found significantly differentially expressed, including 72 downregulated and 184 upregulated genes at the time of progression (Additional file 3: Table S2 and Additional file 4: Table S3).

To analyze if the larger number of patients in the non-response group explained the absence of significant genes we performed a subsampling analysis. This analysis subsampled pairs of non-responders to random groups of 6 patients (100 times) and here we found that the mean number of differentially expressed genes (mean: 2.6; range: 0–33) was approximately 100 times lower than the number of significantly differentially expressed genes found in responders (Additional file 5: Figure S1), indicating the results are not due to differences in sample sizes.

Collectively, we were not able to identify differentially expressed genes between pretreatment samples of responders and non-responders. Furthermore, bevacizumab combination therapy produced a significant impact on the transcriptional changes in responders at time of progression, but only minimal changes in non-responders.

### Functional analysis of transcriptional changes in responders

In contrast to the non-protein coding gene (small nucleolar RNA, H/ACA box 22; *SNORA22*) identified in non-responders, several of the 256 genes identified in responders have been functionally well-characterized in published literature. To identify functional mechanisms related to the gene expression changes in responders, the Molecular Signatures Database (MSigDB) was used to find gene ontologies and gene lists significantly enriched by the up- and down-regulated genes. The top-10 most significantly enriched gene ontologies and gene lists are shown in Additional file 6: Table S4 and Additional file 7: Table S5.

Gene ontology analysis showed that the up-regulated genes are implicated in nervous system development, neuron signaling and neuron differentiation. Down-regulated genes are involved in blood vessel development, collagen metabolism and endodermal differentiation.

Among the gene lists significantly enriched by the upregulated genes are three with high density of CpG-promoters bearing histone H3 trimethylation mark at K27 (H3K27me3). The gene list most significantly enriched by the down-regulated genes characterizes epithelial-mesenchymal transition. Interestingly, the mesenchymal and proneural subtypes defined by Verhaak overlapped significantly with the down-regulated and up-regulated genes, respectively.

Collectively, this analysis shows that responding glioblastomas when progressing express reduced levels of angiogenesis-related genes and higher levels of genes involved in neuronal development and signaling. Furthermore, the gene profiles changed towards a less mesenchymal phenotype and more proneural subtype at progression.

### Dynamical changes in molecular subtype profiles

**Fig. 2:**
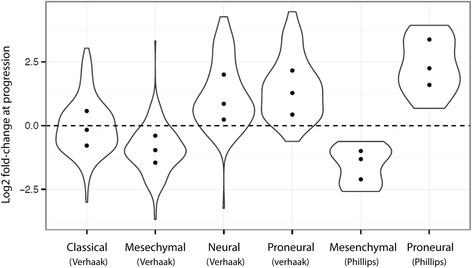
Expression of genes defining molecular subtypes. Paired gene expression fold-changes of genes defining molecular subtypes at the time of progression compared to before initiation of bevacizumab therapy in responding patients. ● indicates the gene expression change according to the 25% percentile of subtype genes. * Modified Phillips classifier used on the AVAglio dataset

To investigate if bevacizumab treatment affects the expression of genes defining the molecular subtypes, gene expression in the paired samples of responders were analyzed according to subtype gene lists [11, 27]. As shown in Fig. 2, we observed that genes defining the Verhaak classical subtype were almost equally up- and downregulated, while the majority of mesenchymal genes were down-regulated at the time of progression. In contrast, most of the neural and proneural genes were upregulated at progression. According to the adapted Phillips classifier all genes of the mesenchymal subtype were down-regulated and all genes of the proneural subtype were up-regulated at progression.

### Ingenuity pathway analysis of transcriptional changes in responders

In order to investigate the structure of possible regulatory networks underlying the significant gene expression changes in responders, we used the IPA. Unlike the enrichment analysis, IPA allows identification of biological networks, including gene relationships and interactions, linked to specific known biological functions or pathways.

First, a canonical pathway analysis was performed to find activated or inhibited pathways. “Integrin signaling” was the only significantly inhibited pathway (Additional file 8: Table S6). Fifteen canonical pathways were found activated and one of these involved “calcium signaling”, while the remaining 14 pathways all included protein kinase C related signaling genes (PRKCB, PRKCE, PRKCZ and others).

**Fig. 3:**
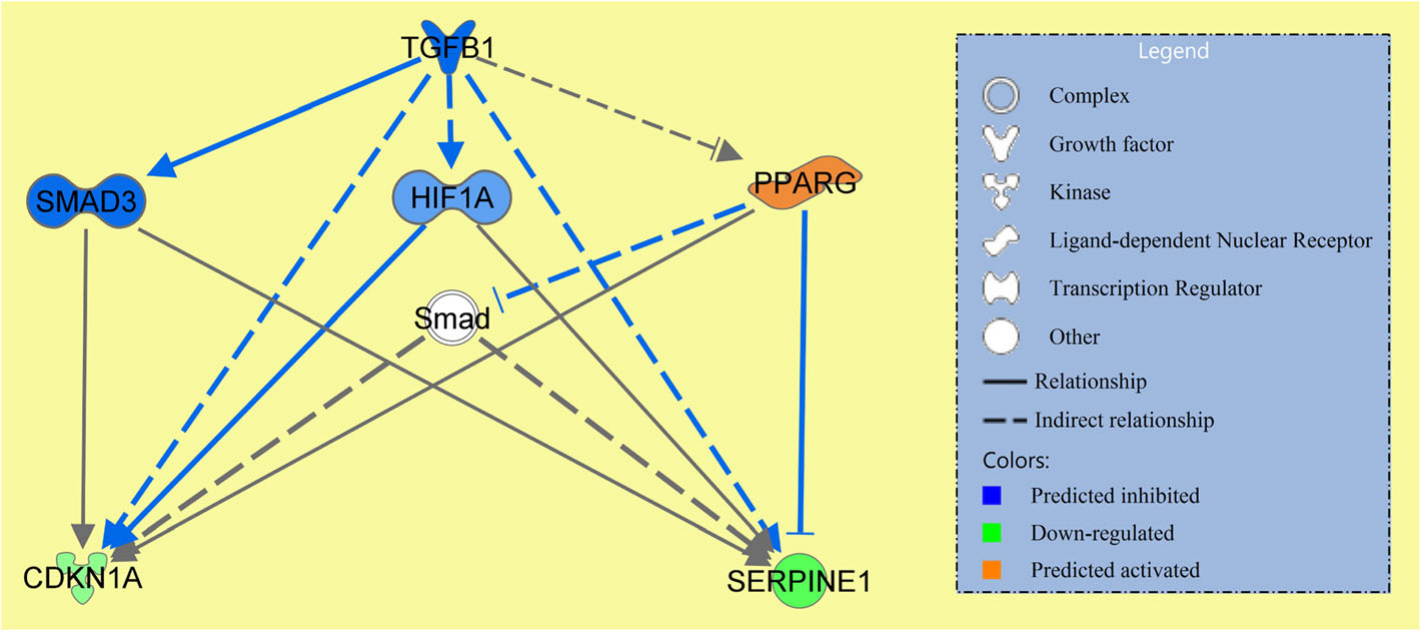
Mechanistic network of inhibited TGF-β1. Generated on the basis of relationships to identified transcriptional changes in responders at the time of progression. The three closest related regulators in the network are SMAD3, HIF1A and PPARG and the two most related downstream molecules are CDKN1A and SERPINE1

The IPA analysis identified 2 activated (estrogen receptor and SPDEF) and 4 inhibited (TGF-β1, SMAD3, ERK and ERRB2) upstream regulators (Additional file 9: Table S7). Out of the 6 upstream regulators, TGF-β1 was the most significant (Z-score = −4.0) and *TGFB1* was the only gene which, based on our RNA-Seq data, trended toward a down-regulation in responders (raw *P* = 0.006; adjusted *P* = 0.15; log2 fold-change = −1.08) while this was not observed in non-responders (raw *P* = 0.57). Consequently, we focused on TGF-β1 and by using the mechanistic network function in IPA, we generated a plausible directional network from TGF-β1 and its closest related upstream regulator molecules. As shown in Fig. 3, this network consisted of two inhibited regulators SMAD3, HIF1A and one activated regulator PPARG, of which *HIF1A* was the only gene trending toward a down-regulation in responders (raw *P* = 0.03; adjusted *P* = 0.36; log2 fold-change = −0.77), while this was not observed in non-responders (raw *P* = 0.51). These upstream-regulators directly or indirectly induce downstream effector molecules involved in cell-cycle check point regulation (CDKN1A) and extracellular matrix remodeling (SERPINE-1). These effector molecules in addition to others, shown in Additional file 10: Figure S2, were predominantly found transcriptionally down-regulated at the time of recurrence, suggesting that TGF-β1 signaling is inactive when tumors progress.

In summary, the pathway analysis showed that protein kinase C signaling was activated in progressing tumors. The analysis found TGF-β1 and HIF1A inhibited and a down-regulated trend was confirmed in the RNA-Seq data. These two up-stream regulators are known to regulate extracellular remodeling and cell-cycle, indicating that responding tumors at progression express reduced extracellular matrix remodeling and increased proliferation.

### Histological changes

To investigate a possible association between the identified transcriptional changes and morphological changes of the tumor, we performed a non-blinded review of hematoxylin-eosin stained pre- and post-treatment samples. However, no gross differences or changes were observed in the amount and morphology of tumor cells, stromal cells, neural/glial cells, blood vessels or architecture of the extracellular matrix. Representative images of two responding glioblastomas are shown in Additional file 11: Figure S3.

## Discussion

In this study of recurrent glioblastoma patients, we performed RNA-Seq on tumor tissue surgically removed before and after bevacizumab combination therapy. In line with others [12], we found no significant differences between pretreatment samples of responders and non-responders. Considering the extreme inter-tumor heterogeneity of glioblastoma and the small sample size, this was not unexpected. Taking this into account, we pre-specified a paired analytical strategy of before and after treatment samples according to treatment response. The results of this analysis reveal significant transcriptional changes in patients responding to bevacizumab while such changes were almost absent in patients not responding. This suggests that non-responding glioblastomas progress chaotically without following any distinct gene expression changes while responding tumors adaptively respond or progress to bevacizumab treatment by means of the same transcriptional changes.

By functional data mining of published literature, we studied the transcriptional changes of responding glioblastomas to uncover potential response and resistance mechanisms to bevacizumab treatment.

First, it is important to acknowledge that the patients underwent relapse surgery 2 months after bevacizumab treatment cessation. This may explain why no morphological changes in the vasculature were observed, as blood vessels can grow and remodel extensively within few weeks [28]. Nevertheless, gene ontology analysis found angiogenesis related genes significantly downregulated at the time of relapse. The reason for these conflicting findings remains unexplained.

The enrichment analysis revealed that up-regulated genes at time of progression were significantly overrepresented by genes involved in neural development and differentiation processes. Furthermore, up-regulated genes were significantly enriched by genes that are known to be up-regulated due to de-methylation of H3K27 promoter regions - a process which is known to be related to decreased activity of Polycomp Repressive Complex 2 (PRC2) during differentiation [29]. Accordingly, epigenetic regulation may be associated with the up-regulated neural differentiation genes. However, no morphological changes were observed in regards to tumor or stromal cells.

It has previously been found that some glioma patients with recurrent disease after non-bevacizumab treatment shift from a proneural tumor into a mesenchymal subtype [30]. Preclinical glioblastoma studies have shown that adaptive resistance to anti-angiogenic agents is characterized by a transition to a mesenchymal phenotype [17, 18]. In contrast, we observed that bevacizumab responding glioblastomas shift into a less mesenchymal and more proneural subtype when progressing.

By using the Ingenuity Pathway Analysis software, we identified TGF-β1 as the most central up-stream regulator associated with the identified gene expression changes. TGF-β1 was found inactivated at time of progression and this was associated with down-regulated extracellular matrix remodeling genes of which several define the mesenchymal subtype signature [27]. This suggests that the shift toward a less mesenchymal phenotype may be related to inactivation of TGF-β1 downstream signaling. In line with this finding, it has been shown in preclinical glioblastoma models that TGF-β signaling induces a mesenchymal shift, while inhibition of TGF-β prevents this shift [31]. Accordingly, the subtypes appear plastic and if the subtypes are representing specific cancer cell lineages, as originally proposed [30], bevacizumab responding glioblastomas may transdifferentiate during progression. Another possibility is that interactions between tumor cells and microenvironment impact subtype classifications, similar to what is seen in epithelial cancers [32]. In this case, bevacizumab induced normalization of the tumor microenvironment and vasculature [33, 34], may change the gene profile accordingly.

Hypoxia has been identified as a central driver of acquired resistance to anti-angiogenic agents in preclinical animal models [16], and hypoxia stimulates secretion of TGF-β which can lead to mesenchymal transition [35]. Thus, one could speculate that reduced hypoxia, as a consequence of bevacizumab-induced vascular normalization, may lead to TGF-β inhibition and reverse mesenchymal transition. Interestingly, and in line with our results, it has recently been found that breast cancer patients responding to bevacizumab demonstrate reduced levels of tumor hypoxia leading to reduced activity of TGF-β [36].

The IPA analysis revealed a significant overrepresentation of genes associated with activated protein kinase C signaling at the time of relapse. This pathway has a central role in tumor-derived VEGF-induced angiogenesis, and in preclinical tumor models protein kinase C inhibitors have shown anti-angiogenic activity [37]. Accordingly, protein kinase C mediated VEGF secretion may induce resistance to bevacizumab and may serve as a target in bevacizumab responding glioblastoma patients.

Our study presented a few limitations. The small number of highly selected patients may or may not have introduced selection bias. The lack of paired glioblastomas treated without bevacizumab containing regimens make it difficult to interpret whether the observed effects are related to bevacizumab response or treatment response in general. In this context, the exploratory results have to be carefully interpreted taking these limitations into account. Nevertheless, the baseline patient/sample characteristics and gene profiles did not differ between responding and non-responding patients and the pre-analytical design of the study make it less likely that the observed changes are independent of response to bevacizumab combination therapy.

## Conclusions

To our knowledge this is the first study to demonstrate that bevacizumab combination treatment has a significant impact on transcriptional changes in a paired analysis of responding glioblastoma patients. Such changes were minimal in patients not responding. In conclusion, we hypothesize that the identified adaptive changes of bevacizumab responding glioblastomas are related to response or resistance mechanisms. If validated, these data may prove valuable for identification of new and more efficient bevacizumab combination regimens.

## Additional files

Additional file 1: Supplementary methods Analysis of study dataset: Mapping and quantification, Filtering and differential expression, Gene-set analysis (DOCX 26 kb)

Additional file 2: Table S1. Differentially expressed gene in non-responders (1 gene) (DOCX 14 kb)

Additional file 3: Table S2. Down-regulated genes in responders (72 genes) (DOCX 21 kb)

Additional file 4: Table S3. Up-regulated genes in responders (184 genes) (DOCX 32 kb)

Additional file 5: Figure S1. Subsampling analysis. This analysis subsampled pairs of non-responders (Comparison 3) to random groups of 6 patients 100 times. Comparison 1 and Comparison 3 shows the number of differentially expressed genes in the paired comparison analysis of responders and non-responders, respectively (DOCX 51 kb)

Additional file 6: Table S4. Gene set enrichment analysis of up-regulated genes (DOCX 17 kb)

Additional file 7: Table S5. Gene set enrichment analysis of down-regulated genes (DOCX 18 kb)

Additional file 8: Table S6. Ingenuity Pathway Analysis of activated and inhibited canonical pathways (DOCX 18 kb)

Additional file 9: Table S7. Ingenuity Pathway Analysis of activated and inhibited up-stream regulators (DOCX 15 kb)

Additional file 10: Figure S2. Mechanistic network of inhibited TGF-β1 and the three most interconnected regulators (SMAD3, HIF1A and PPARG) of downstream molecules identified differentially expressed in responders at the time of progression (DOCX 122 kb)

Additional file 11: Figure S3. Hematoxylin-eosin staining of two representative responding glioblastomas before and after bevacizumab therapy (DOCX 2297 kb)

## Abbreviations

CDKN1A: Cyclin-dependent kinase inhibitor 1A
CR: Complete response
ERK: Extracellular signal-regulated kinase
ERRB2: erb-b2 receptor tyrosine kinase 2
FDR: False discovery rate
FFPE: Formalin-fixed paraffin embedded
FPKM: Fragments per kilobase per million reads
H3K27me3: Histone H3 thimethylation mark at K27
HIF1A: Hypoxia-inducible factor 1-alpha
IPA: Ingenuity pathway analysis
MRI: Magnetic resonance imaging
MSigDB: Molecular signatures database
PD: Progressive disease
PPARG: Peroxisome proliferator-activated receptor gamma
PR: Partial response
PRC2: Polycomp repressive complex 2
PRKCB: Protein kinase C, beta
PRKCE: Protein kinase C, epsilon
PRKCZ: Protein kinase C, zeta
RANO: Response assessment in neuro-oncology criteria
RNA-seq: RNA sequencing
SD: Stable disease
SERPINE-1: Serine protease inhibitor 1
SPDEF: Sam pointed domain containing ETS transcription factor
TGF-β1: Transforming growth factor beta 1
VEGF: Vascular endothelial growth factor
