# Supplementary material for: Transcriptional changes induced by bevacizumab combination therapy in responding and non-responding recurrent glioblastoma patients
Source: BMC Cancer. 2017 Apr 18;17:278. doi: 10.1186/s12885-017-3251-3 (PMC5395849; doi:10.1186/s12885-017-3251-3)
Supplement: Supplementary file 1 — Supplementary methods Analysis of study dataset: Mapping and quantification, Filtering and differential expression, Gene-set analysis (DOCX 26 kb) [file 12885_2017_3251_MOESM1_ESM.docx]

# Supplementary methods

## Analysis of study dataset

## Mapping and quantification

The Raw sequencing data was trimmed with Trimmomatic (v 0.32) with the parameters: ILLUMINACLIP:fastqc.fa:2:30:10 HEADCROP:X LEADING:22 SLIDINGWINDOW:4:22 MINLEN:25 where fastqc.fa is a fasta file containing all the adapters that are distributed with FastQC v0.11.2 and where X is an individual number (mean=8, range=1-21) to remove the reverse transcriptase (RT) bias in the individual libaries.^1^ The trimmed data was mapped to the human genome (hg19) using TopHat2 (v2.0.9) using the parameters --b2-very-sensitive --library-type=fr-firststrand and supplying the Enslemble (release 66) GTF file to the --GTF argument. The mapped reads were summarized into gene counts using the featureCounts() function,^2^ from the Rsubread R package (V1.16.1) using the same GTF file as annotation and default parameters apart from isPairedEnd=TRUE, minReadOverlap=10 and the paired-end orientation described above.^3^

## Filtering and differential expression

Libraries with less than 10% of genes having more than 15 fragments were discarded (n=6). Only genes with at least 10 fragments and an abundance of at least 3 Fragments Per Kilobase pr Million reads (FPKM) in at least 5 libraries in any of the 4 patient-groups were kept for further analysis (n=15630).

Differential expression analysis was performed with edgeR (v 3.12.0)

using glmLRT() after RLE normalization and dispersion estimation with estimateGLMCommonDisp(), estimateGLMTrendedDisp() and estimateGLMTagwiseDisp().^4^ p-values were corrected for multiple testing using the False Discovery Rate (FDR) approach and genes with a q-value (FDR corrected p-value) < 0.05 were considered significant.

## Gene-set analysis

Gene Ontology (GO) gene-sets were downloaded (6^th^ Jan 2016) from The European Bioinformatics Institute’s official Gene Ontology mirror. GO-terms from the 5th level (distance = 6) of the herarical GO-term tree was used. MSigDB’s gene sets c2, c3, c6 and H was downloaded from (<http://bioinf.wehi.edu.au/software/MSigDB/>).^5^ All gene id’s were converted to ensemble gene ids using the biomaRt R package.^6^ The enrichment analysis was done using a Fisher’s exact test and p-values were FDR corrected. Genes-sets with a q-value < 0.05 were considered significant.

Reference List

(1) Li S, Tighe SW, Nicolet CM et al. Multi-platform assessment of transcriptome profiling using RNA-seq in the ABRF next-generation sequencing study. *Nat Biotechnol* 2014;32:915-925.

(2) Liao Y, Smyth GK, Shi W. featureCounts: an efficient general purpose program for assigning sequence reads to genomic features. *Bioinformatics* 2014;30:923-930.

(3) Liao Y, Smyth GK, Shi W. The Subread aligner: fast, accurate and scalable read mapping by seed-and-vote. *Nucleic Acids Res* 2013;41:e108.

(4) Robinson MD, McCarthy DJ, Smyth GK. edgeR: a Bioconductor package for differential expression analysis of digital gene expression data. *Bioinformatics* 2010;26:139-140.

(5) Liberzon A, Subramanian A, Pinchback R, Thorvaldsdottir H, Tamayo P, Mesirov JP. Molecular signatures database (MSigDB) 3.0. *Bioinformatics* 2011;27:1739-1740.

(6) Durinck S, Moreau Y, Kasprzyk A et al. BioMart and Bioconductor: a powerful link between biological databases and microarray data analysis. *Bioinformatics* 2005;21:3439-3440.
