# Supplementary material for: Transcriptional changes induced by bevacizumab combination therapy in responding and non-responding recurrent glioblastoma patients
Source: BMC Cancer. 2017 Apr 18;17:278. doi: 10.1186/s12885-017-3251-3 (PMC5395849; doi:10.1186/s12885-017-3251-3)
Supplement: Supplementary file 2 — Differentially expressed gene in non-responders (1 gene) (DOCX 14 kb) [file 12885_2017_3251_MOESM2_ESM.docx]

## Table S1 – Differentially expressed gene in non-responders (1 gene)

| Gene ID | Gene Name | Adj. *P*-value | Log2FC |
| --- | --- | --- | --- |
| ENSG00000206634 | SNORA22 | 0.0004 | 1.68 |
