# Supplementary material for: Transcriptional changes induced by bevacizumab combination therapy in responding and non-responding recurrent glioblastoma patients
Source: BMC Cancer. 2017 Apr 18;17:278. doi: 10.1186/s12885-017-3251-3 (PMC5395849; doi:10.1186/s12885-017-3251-3)
Supplement: Supplementary file 3 — Down-regulated genes in responders (72 genes) (DOCX 21 kb) [file 12885_2017_3251_MOESM3_ESM.docx]

## Table S2 - Down-regulated genes in responders (72 genes)

| Gene ID | Gene Name | Adj. *P*-value | Log2FC |
| --- | --- | --- | --- |
| ENSG00000168542 | *COL3A1* | 1.8E-07 | -2.82 |
| ENSG00000108821 | *COL1A1* | 3.1E-06 | -3.01 |
| ENSG00000106366 | *SERPINE1* | 6.6E-06 | -2.59 |
| ENSG00000163359 | *COL6A3* | 6.0E-05 | -2.59 |
| ENSG00000148848 | *ADAM12* | 0.0003 | -2.44 |
| ENSG00000130635 | *COL5A1* | 0.0005 | -2.33 |
| ENSG00000187498 | *COL4A1* | 0.0006 | -2.47 |
| ENSG00000164692 | *COL1A2* | 0.0006 | -2.23 |
| ENSG00000187678 | *SPRY4* | 0.0006 | -2.18 |
| ENSG00000110492 | *MDK* | 0.0011 | -2.21 |
| ENSG00000134871 | *COL4A2* | 0.0025 | -2.12 |
| ENSG00000137801 | *THBS1* | 0.0034 | -1.99 |
| ENSG00000091136 | *LAMB1* | 0.0040 | -1.70 |
| ENSG00000112769 | *LAMA4* | 0.0042 | -1.67 |
| ENSG00000107796 | *ACTA2* | 0.0051 | -1.97 |
| ENSG00000018280 | *SLC11A1* | 0.0065 | -2.01 |
| ENSG00000146674 | *IGFBP3* | 0.0071 | -2.29 |
| ENSG00000132530 | *XAF1* | 0.0071 | -1.88 |
| ENSG00000150636 | *CCDC102B* | 0.0089 | -2.97 |
| ENSG00000181104 | *F2R* | 0.0010 | -1.73 |
| ENSG00000010327 | *STAB1* | 0.0106 | -1.45 |
| ENSG00000115414 | *FN1* | 0.0108 | -1.80 |
| ENSG00000157227 | *MMP14* | 0.0113 | -1.74 |
| ENSG00000163430 | *FSTL1* | 0.0115 | -1.37 |
| ENSG00000261295 | *RP11-524D16__A.3.1* | 0.0129 | -3.53 |
| ENSG00000149948 | *HMGA2* | 0.0146 | -2.28 |
| ENSG00000174807 | *CD248* | 0.0146 | -2.13 |
| ENSG00000120318 | *ARAP3* | 0.0155 | -2.10 |
| ENSG00000226053 | *RP5-1070A16.1.1* | 0.0175 | -5.83 |
| ENSG00000103196 | *CRISPLD2* | 0.0175 | -2.79 |
| ENSG00000061337 | *LZTS1* | 0.0185 | -1.87 |
| ENSG00000120708 | *TGFBI* | 0.0187 | -1.94 |
| ENSG00000082074 | *FYB* | 0.0187 | -1.86 |
| ENSG00000163694 | *RBM47* | 0.0187 | -1.74 |
| ENSG00000141753 | *IGFBP4* | 0.0187 | -1.60 |
| ENSG00000161638 | *ITGA5* | 0.0197 | -1.81 |
| ENSG00000182718 | *ANXA2* | 0.0204 | -1.37 |
| ENSG00000184060 | *ADAP2* | 0.0221 | -1.92 |
| ENSG00000135424 | *ITGA7* | 0.0229 | -1.65 |
| ENSG00000159216 | *RUNX1* | 0.0230 | -1.52 |
| ENSG00000150551 | *LYPD1* | 0.0232 | -2.22 |
| ENSG00000196083 | *IL1RAP* | 0.0232 | -1.96 |
| ENSG00000111252 | *SH2B3* | 0.0232 | -1.37 |
| ENSG00000147614 | *ATP6V0D2* | 0.0233 | -6.63 |
| ENSG00000183486 | *MX2* | 0.0233 | -3.03 |
| ENSG00000124762 | *CDKN1A* | 0.0233 | -1.71 |
| ENSG00000186470 | *BTN3A2* | 0.0240 | -2.07 |
| ENSG00000142798 | *HSPG2* | 0.0240 | -1.66 |
| ENSG00000010610 | *CD4* | 0.0241 | -1.67 |
| ENSG00000177469 | *PTRF* | 0.0269 | -1.36 |
| ENSG00000076706 | *MCAM* | 0.0279 | -1.47 |
| ENSG00000106991 | *ENG* | 0.0287 | -1.70 |
| ENSG00000173068 | *BNC2* | 0.0316 | -2.55 |
| ENSG00000149257 | *SERPINH1* | 0.0321 | -1.68 |
| ENSG00000221818 | *EBF2* | 0.0326 | -4.49 |
| ENSG00000213949 | *ITGA1* | 0.0326 | -2.01 |
| ENSG00000134013 | *LOXL2* | 0.0333 | -1.80 |
| ENSG00000130429 | *ARPC1B* | 0.0350 | -2.06 |
| ENSG00000124813 | *RUNX2* | 0.0353 | -1.75 |
| ENSG00000173369 | *C1QB* | 0.0357 | -1.47 |
| ENSG00000182492 | *BGN* | 0.0367 | -1.43 |
| ENSG00000143226 | *FCGR2A* | 0.0392 | -1.35 |
| ENSG00000261468 | *RP11-1024P17.1.1* | 0.0426 | -5.36 |
| ENSG00000186407 | *CD300E* | 0.0427 | -3.18 |
| ENSG00000066336 | *SPI1* | 0.0433 | -1.57 |
| ENSG00000102265 | *TIMP1* | 0.0437 | -1.80 |
| ENSG00000130052 | *STARD8* | 0.0444 | -2.80 |
| ENSG00000060138 | *CSDA* | 0.0452 | -1.51 |
| ENSG00000137745 | *MMP13* | 0.0466 | -5.95 |
| ENSG00000100292 | *HMOX1* | 0.0466 | -1.94 |
| ENSG00000254369 | *RP1-170O19.2.1* | 0.0493 | -6.55 |
| ENSG00000128641 | *MYO1B* | 0.0496 | -1.80 |
