# Supplementary material for: Transcriptional changes induced by bevacizumab combination therapy in responding and non-responding recurrent glioblastoma patients
Source: BMC Cancer. 2017 Apr 18;17:278. doi: 10.1186/s12885-017-3251-3 (PMC5395849; doi:10.1186/s12885-017-3251-3)
Supplement: Supplementary file 4 — Up-regulated genes in responders (184 genes) (DOCX 32 kb) [file 12885_2017_3251_MOESM4_ESM.docx]

## Table S3 - Up-regulated genes in responders (184 genes)

| Gene ID | Gene Name | Adj. *P*-value | Log2FC |
| --- | --- | --- | --- |
| ENSG00000132692 | *BCAN* | 3.8E-05 | 3.66 |
| ENSG00000175161 | *CADM2* | 4.5E-05 | 2.20 |
| ENSG00000104888 | *SLC17A7* | 0.0004 | 6.35 |
| ENSG00000183248 | *AC010336.1* | 0.0005 | 2.40 |
| ENSG00000148798 | *INA* | 0.0006 | 6.04 |
| ENSG00000175497 | *DPP10* | 0.0006 | 4.75 |
| ENSG00000104833 | *TUBB4A* | 0.0006 | 4.12 |
| ENSG00000112290 | *WASF1* | 0.0006 | 3.33 |
| ENSG00000261786 | *RP4-555D20.2.1* | 0.0010 | 3.54 |
| ENSG00000152932 | *RAB3C* | 0.0011 | 4.93 |
| ENSG00000165443 | *PHYHIPL* | 0.0011 | 2.28 |
| ENSG00000177511 | *ST8SIA3* | 0.0014 | 5.10 |
| ENSG00000100167 | *SEPT3* | 0.0015 | 3.73 |
| ENSG00000008086 | *CDKL5* | 0.0023 | 3.38 |
| ENSG00000077279 | *DCX* | 0.0023 | 3.05 |
| ENSG00000198910 | *L1CAM* | 0.0024 | 4.23 |
| ENSG00000166897 | *ELFN2* | 0.0030 | 3.39 |
| ENSG00000124507 | *PACSIN1* | 0.0040 | 5.23 |
| ENSG00000008056 | *SYN1* | 0.0040 | 4.97 |
| ENSG00000104435 | *STMN2* | 0.0040 | 4.71 |
| ENSG00000132639 | *SNAP25* | 0.0040 | 3.98 |
| ENSG00000107758 | *PPP3CB* | 0.0040 | 2.18 |
| ENSG00000173786 | *CNP* | 0.0040 | 1.95 |
| ENSG00000257151 | *RP11-701H24.2.1* | 0.0040 | 1.68 |
| ENSG00000182195 | *LDOC1* | 0.0040 | 2.79 |
| ENSG00000107105 | *ELAVL2* | 0.0040 | 3.80 |
| ENSG00000168490 | *PHYHIP* | 0.0041 | 4.89 |
| ENSG00000179915 | *NRXN1* | 0.0041 | 2.46 |
| ENSG00000157152 | *SYN2* | 0.0044 | 5.61 |
| ENSG00000162188 | *GNG3* | 0.0047 | 5.14 |
| ENSG00000105649 | *RAB3A* | 0.0047 | 4.48 |
| ENSG00000164061 | *BSN* | 0.0049 | 3.62 |
| ENSG00000145920 | *CPLX2* | 0.0049 | 4.80 |
| ENSG00000176381 | *PRR18* | 0.0050 | 6.45 |
| ENSG00000167654 | *ATCAY* | 0.0050 | 3.54 |
| ENSG00000070087 | *PFN2* | 0.0065 | 1.53 |
| ENSG00000133169 | *BEX1* | 0.0070 | 3.77 |
| ENSG00000177807 | *KCNJ10* | 0.0074 | 4.09 |
| ENSG00000237289 | *CKMT1B* | 0.0086 | 5.46 |
| ENSG00000136531 | *SCN2A* | 0.0086 | 2.57 |
| ENSG00000008300 | *CELSR3* | 0.0086 | 1.98 |
| ENSG00000047597 | *XK* | 0.0088 | 6.02 |
| ENSG00000168314 | *MOBP* | 0.0088 | 2.73 |
| ENSG00000171132 | *PRKCE* | 0.0091 | 2.36 |
| ENSG00000130540 | *SULT4A1* | 0.0094 | 3.81 |
| ENSG00000179292 | *TMEM151A* | 0.0095 | 5.12 |
| ENSG00000101210 | *EEF1A2* | 0.0095 | 4.18 |
| ENSG00000114646 | *CSPG5* | 0.0095 | 2.11 |
| ENSG00000169851 | *PCDH7* | 0.0096 | 2.98 |
| ENSG00000074317 | *SNCB* | 0.0103 | 5.91 |
| ENSG00000166342 | *NETO1* | 0.0103 | 4.13 |
| ENSG00000184611 | *KCNH7* | 0.0107 | 5.00 |
| ENSG00000136928 | *GABBR2* | 0.0107 | 3.65 |
| ENSG00000130287 | *NCAN* | 0.0107 | 3.47 |
| ENSG00000078018 | *MAP2* | 0.0107 | 2.27 |
| ENSG00000166165 | *CKB* | 0.0107 | 2.16 |
| ENSG00000197959 | *DNM3* | 0.0108 | 1.52 |
| ENSG00000254377 | *RP11-32K4.2.1* | 0.0115 | 4.49 |
| ENSG00000171450 | *CDK5R2* | 0.0115 | 4.11 |
| ENSG00000154146 | *NRGN* | 0.0120 | 4.22 |
| ENSG00000018625 | *ATP1A2* | 0.0123 | 1.96 |
| ENSG00000105409 | *ATP1A3* | 0.0123 | 4.50 |
| ENSG00000104112 | *SCG3* | 0.0145 | 2.43 |
| ENSG00000166257 | *SCN3B* | 0.0145 | 3.00 |
| ENSG00000176884 | *GRIN1* | 0.0146 | 4.65 |
| ENSG00000136854 | *STXBP1* | 0.0149 | 2.98 |
| ENSG00000187189 | *TSPYL4* | 0.0149 | 1.79 |
| ENSG00000173320 | *STOX2* | 0.0150 | 1.70 |
| ENSG00000168280 | *KIF5C* | 0.0155 | 2.10 |
| ENSG00000084628 | *NKAIN1* | 0.0156 | 3.61 |
| ENSG00000126861 | *OMG* | 0.0174 | 4.17 |
| ENSG00000157064 | *NMNAT2* | 0.0174 | 2.54 |
| ENSG00000166448 | *TMEM130* | 0.0175 | 3.94 |
| ENSG00000100146 | *SOX10* | 0.0175 | 2.76 |
| ENSG00000186231 | *KLHL32* | 0.0175 | 4.10 |
| ENSG00000145864 | *GABRB2* | 0.0179 | 4.31 |
| ENSG00000177301 | *KCNA2* | 0.0179 | 3.95 |
| ENSG00000103034 | *NDRG4* | 0.0179 | 1.93 |
| ENSG00000118160 | *SLC8A2* | 0.0187 | 4.04 |
| ENSG00000099822 | *HCN2* | 0.0187 | 3.43 |
| ENSG00000109107 | *ALDOC* | 0.0187 | 3.22 |
| ENSG00000165152 | *C9orf125* | 0.0197 | 3.66 |
| ENSG00000144290 | *SLC4A10* | 0.0197 | 4.42 |
| ENSG00000197177 | *GPR123* | 0.0197 | 4.18 |
| ENSG00000172995 | *ARPP21* | 0.0197 | 2.71 |
| ENSG00000101445 | *PPP1R16B* | 0.0201 | 2.61 |
| ENSG00000198794 | *SCAMP5* | 0.0201 | 2.41 |
| ENSG00000243156 | *MICAL3* | 0.0201 | 1.42 |
| ENSG00000125648 | *SLC25A23* | 0.0201 | 1.31 |
| ENSG00000105613 | *MAST1* | 0.0208 | 3.55 |
| ENSG00000224189 | *AC009336.23.1* | 0.0221 | 5.47 |
| ENSG00000188191 | *PRKAR1B* | 0.0221 | 2.60 |
| ENSG00000084731 | *KIF3C* | 0.0221 | 1.92 |
| ENSG00000075340 | *ADD2* | 0.0221 | 1.55 |
| ENSG00000185046 | *ANKS1B* | 0.0224 | 1.87 |
| ENSG00000165388 | *ZNF488* | 0.0229 | 4.90 |
| ENSG00000155980 | *KIF5A* | 0.0229 | 3.29 |
| ENSG00000113327 | *GABRG2* | 0.0230 | 5.57 |
| ENSG00000130558 | *OLFM1* | 0.0232 | 4.28 |
| ENSG00000109654 | *TRIM2* | 0.0232 | 1.52 |
| ENSG00000159409 | *CELF3* | 0.0233 | 4.10 |
| ENSG00000168243 | *GNG4* | 0.0233 | 3.52 |
| ENSG00000008735 | *MAPK8IP2* | 0.0233 | 3.18 |
| ENSG00000171617 | *ENC1* | 0.0233 | 2.36 |
| ENSG00000196361 | *ELAVL3* | 0.0233 | 2.35 |
| ENSG00000167123 | *CERCAM* | 0.0238 | 1.73 |
| ENSG00000178233 | *TMEM151B* | 0.0240 | 3.08 |
| ENSG00000173898 | *SPTBN2* | 0.0240 | 2.81 |
| ENSG00000163032 | *VSNL1* | 0.0240 | 3.83 |
| ENSG00000122966 | *CIT* | 0.0249 | 1.97 |
| ENSG00000145087 | *STXBP5L* | 0.0252 | 2.99 |
| ENSG00000171532 | *NEUROD2* | 0.0253 | 4.89 |
| ENSG00000067715 | *SYT1* | 0.0263 | 3.98 |
| ENSG00000197971 | *MBP* | 0.0263 | 3.14 |
| ENSG00000054356 | *PTPRN* | 0.0263 | 2.34 |
| ENSG00000134709 | *HOOK1* | 0.0265 | 5.09 |
| ENSG00000127585 | *FBXL16* | 0.0268 | 2.81 |
| ENSG00000073464 | *CLCN4* | 0.0268 | 2.01 |
| ENSG00000165868 | *HSPA12A* | 0.0273 | 2.43 |
| ENSG00000196338 | *NLGN3* | 0.0283 | 1.40 |
| ENSG00000259969 | *RP11-999E24.3.1* | 0.0287 | 5.43 |
| ENSG00000107295 | *SH3GL2* | 0.0287 | 4.27 |
| ENSG00000019505 | *SYT13* | 0.0287 | 4.19 |
| ENSG00000164742 | *ADCY1* | 0.0287 | 1.94 |
| ENSG00000260918 | *RP11-731J8.2.1* | 0.0293 | 3.87 |
| ENSG00000151150 | *ANK3* | 0.0293 | 2.24 |
| ENSG00000008277 | *ADAM22* | 0.0293 | 1.37 |
| ENSG00000123560 | *PLP1* | 0.0297 | 3.17 |
| ENSG00000221823 | *PPP3R1* | 0.0297 | 1.42 |
| ENSG00000020129 | *NCDN* | 0.0297 | 2.10 |
| ENSG00000170579 | *DLGAP1* | 0.0302 | 3.10 |
| ENSG00000112379 | *KIAA1244* | 0.0302 | 1.60 |
| ENSG00000149927 | *DOC2A* | 0.0306 | 3.59 |
| ENSG00000250686 | *RP1-240B8.3.1* | 0.0326 | 5.25 |
| ENSG00000128872 | *TMOD2* | 0.0332 | 1.56 |
| ENSG00000113763 | *UNC5A* | 0.0334 | 3.26 |
| ENSG00000006116 | *CACNG3* | 0.0342 | 4.44 |
| ENSG00000110786 | *PTPN5* | 0.0342 | 3.82 |
| ENSG00000100095 | *SEZ6L* | 0.0342 | 3.22 |
| ENSG00000154917 | *RAB6B* | 0.0342 | 2.06 |
| ENSG00000187391 | *MAGI2* | 0.0342 | 1.43 |
| ENSG00000172508 | *CARNS1* | 0.0346 | 2.48 |
| ENSG00000175874 | *CREG2* | 0.0354 | 3.65 |
| ENSG00000177108 | *ZDHHC22* | 0.0357 | 3.44 |
| ENSG00000157087 | *ATP2B2* | 0.0357 | 3.20 |
| ENSG00000255571 | *CTD-2335A18.1.1* | 0.0357 | 2.78 |
| ENSG00000110881 | *ACCN2* | 0.0357 | 2.34 |
| ENSG00000144230 | *GPR17* | 0.0370 | 4.44 |
| ENSG00000126950 | *TMEM35* | 0.0370 | 4.38 |
| ENSG00000123119 | *NECAB1* | 0.0380 | 3.37 |
| ENSG00000156298 | *TSPAN7* | 0.0388 | 1.90 |
| ENSG00000150672 | *DLG2* | 0.0391 | 2.07 |
| ENSG00000106123 | *EPHB6* | 0.0403 | 3.73 |
| ENSG00000087258 | *GNAO1* | 0.0403 | 3.07 |
| ENSG00000050030 | *KIAA2022* | 0.0403 | 2.52 |
| ENSG00000067606 | *PRKCZ* | 0.0411 | 2.52 |
| ENSG00000122584 | *NXPH1* | 0.0420 | 4.35 |
| ENSG00000078328 | *RBFOX1* | 0.0426 | 3.13 |
| ENSG00000196090 | *PTPRT* | 0.0426 | 2.97 |
| ENSG00000166501 | *PRKCB* | 0.0426 | 2.38 |
| ENSG00000136960 | *ENPP2* | 0.0433 | 2.40 |
| ENSG00000172137 | *CALB2* | 0.0433 | 5.88 |
| ENSG00000099308 | *MAST3* | 0.0437 | 1.84 |
| ENSG00000154027 | *AK5* | 0.0437 | 3.24 |
| ENSG00000110076 | *NRXN2* | 0.0437 | 1.97 |
| ENSG00000076826 | *CAMSAP3* | 0.0439 | 4.53 |
| ENSG00000185760 | *KCNQ5* | 0.0442 | 2.87 |
| ENSG00000224223 | *GS1-18A18.1.1* | 0.0455 | 5.94 |
| ENSG00000180440 | *SERTM1* | 0.0460 | 4.64 |
| ENSG00000130294 | *KIF1A* | 0.0461 | 3.08 |
| ENSG00000110400 | *PVRL1* | 0.0466 | 2.18 |
| ENSG00000128594 | *LRRC4* | 0.0466 | 2.17 |
| ENSG00000139915 | *MDGA2* | 0.0466 | 4.29 |
| ENSG00000114279 | *FGF12* | 0.0466 | 3.41 |
| ENSG00000163539 | *CLASP2* | 0.0466 | 1.65 |
| ENSG00000167971 | *CASKIN1* | 0.0485 | 3.12 |
| ENSG00000123091 | *RNF11* | 0.0485 | 1.55 |
| ENSG00000118276 | *B4GALT6* | 0.0486 | 4.08 |
| ENSG00000122733 | *KIAA1045* | 0.0493 | 3.90 |
| ENSG00000184144 | *CNTN2* | 0.0493 | 2.94 |
| ENSG00000185742 | *C11orf87* | 0.0496 | 3.89 |
| ENSG00000007237 | *GAS7* | 0.0496 | 1.29 |
| ENSG00000018236 | *CNTN1* | 0.0496 | 3.66 |
| ENSG00000186472 | *PCLO* | 0.0496 | 2.97 |
