# Supplementary material for: Transcriptional changes induced by bevacizumab combination therapy in responding and non-responding recurrent glioblastoma patients
Source: BMC Cancer. 2017 Apr 18;17:278. doi: 10.1186/s12885-017-3251-3 (PMC5395849; doi:10.1186/s12885-017-3251-3)
Supplement: Supplementary file 5 — Subsampling analysis. This analysis subsampled pairs of non-responders (Comparison 3) to random groups of 6 patients 100 times. Comparison 1 and Comparison 3 shows the number of differentially expressed genes in the paired comparison analysis of responders and non-responders, respectively (DOCX 51 kb) [file 12885_2017_3251_MOESM5_ESM.docx]

Figure S1. Subsampling analysis. This analysis subsampled pairs of non-responders (Comparison 3) to random groups of 6 patients 100 times. Comparison 1 and Comparison 3 shows the number of differentially expressed genes in the paired comparison analysis of responders and non-responders, respectively.
