# Supplementary material for: Transcriptional changes induced by bevacizumab combination therapy in responding and non-responding recurrent glioblastoma patients
Source: BMC Cancer. 2017 Apr 18;17:278. doi: 10.1186/s12885-017-3251-3 (PMC5395849; doi:10.1186/s12885-017-3251-3)
Supplement: Supplementary file 6 — Gene set enrichment analysis of up-regulated genes (DOCX 17 kb) [file 12885_2017_3251_MOESM6_ESM.docx]

## Table S4 – Gene set enrichment analysis of up-regulated genes

| **GeneSet_ID (Gene Ontology)** | ***P*-Value** | **Odds ratio** | **False discovery rate** |
| --- | --- | --- | --- |
| GO_nervous_system_development | 7.76E-23 | 5.1 | 3.18E-19 |
| GO_modulation_of_synaptic_transmission | 1.55E-18 | 12.5 | 2.55E-15 |
| GO_neuron_projection_development | 3.71E-16 | 5.2 | 3.81E-13 |
| GO_neurogenesis | 2.05E-15 | 4.3 | 1.87E-12 |
| GO_neuron_differentiation | 4.67E-15 | 4.4 | 3.83E-12 |
| GO_cognition | 3.94E-14 | 10.6 | 2.49E-11 |
| GO_neurotransmitter_transport | 3.00E-13 | 12.1 | 1.54E-10 |
| GO_cell_development | 2.61E-12 | 3.5 | 1.13E-09 |
| GO_synaptic_vesicle_localization | 3.78E-12 | 14.0 | 1.55E-09 |
| GO_neuron_projection_morphogenesis | 1.13E-11 | 4.6 | 3.77E-09 |

| **GeneSet_ID (Gene lists)** | ***P*-Value** | **Odds ratio** | **False discovery rate** |
| --- | --- | --- | --- |
| c2_MIKKELSEN_MEF_HCP_WITH_H3K27ME3 | 9.38E-33 | 16.7 | 7.70E-29 |
| c2_BLALOCK_ALZHEIMERS_DISEASE_DN | 8.54E-21 | 5.6 | 1.75E-17 |
| c2_VERHAAK_GLIOBLASTOMA_PRONEURAL | 7.10E-21 | 17.2 | 1.75E-17 |
| c2_YOSHIMURA_MAPK8_TARGETS_UP | 4.04E-18 | 5.8 | 5.54E-15 |
| c3_V$NRSF_01 | 6.06E-17 | 28.1 | 7.11E-14 |
| c6_KRAS.KIDNEY_UP.V1_UP | 8.70E-15 | 15.2 | 6.50E-12 |
| c2_MIKKELSEN_MCV6_HCP_WITH_H3K27ME3 | 3.45E-14 | 9.3 | 2.36E-11 |
| c2_REACTOME_NEURONAL_SYSTEM | 7.66E-14 | 10.2 | 4.49E-11 |
| c2_KIM_ALL_DISORDERS_CALB1_CORR_UP | 1.61E-13 | 6.0 | 8.81E-11 |
| c2_BENPORATH_ES_WITH_H3K27ME3 | 8.83E-13 | 5.1 | 4.27E-10 |
