# Supplementary material for: Transcriptional changes induced by bevacizumab combination therapy in responding and non-responding recurrent glioblastoma patients
Source: BMC Cancer. 2017 Apr 18;17:278. doi: 10.1186/s12885-017-3251-3 (PMC5395849; doi:10.1186/s12885-017-3251-3)
Supplement: Supplementary file 7 — Gene set enrichment analysis of down-regulated genes (DOCX 18 kb) [file 12885_2017_3251_MOESM7_ESM.docx]

## Table S5 – Gene set enrichment analysis of down-regulated genes

| **GeneSet_ID (Gene Ontology)** | ***P*-Value** | **Odds ratio** | **False discovery rate** |
| --- | --- | --- | --- |
| GO_blood_vessel_development | 2.02E-19 | 17.4 | 2.37E-16 |
| GO_vasculature_development | 5.70E-19 | 16.5 | 5.85E-16 |
| GO_cardiovascular_system_development | 9.18E-16 | 11.1 | 6.28E-13 |
| GO_circulatory_system_development | 9.18E-16 | 11.1 | 6.28E-13 |
| GO_collagen_metabolic_process | 2.79E-13 | 35.3 | 1.04E-10 |
| GO_endodermal_cell_differentiation | 2.89E-12 | 71.7 | 9.14E-10 |
| GO_endoderm_formation | 9.29E-12 | 60.5 | 2.54E-09 |
| GO_skeletal_system_development | 1.52E-11 | 11.8 | 4.02E-09 |
| GO_collagen_fibril_organization | 2.45E-10 | 57.5 | 4.47E-08 |
| GO_cartilage_development | 6.97E-10 | 19.4 | 1.15E-07 |

| **GeneSet_ID (Gene list)** | ***P*-Value** | **Odds ratio** | **False discovery rate** |
| --- | --- | --- | --- |
| ch_HALLMARK_EPITHELIAL_MESENCHYMAL_TRANSITION | 1.22E-25 | 41.5 | 1.00E-21 |
| c2_VERHAAK_GLIOBLASTOMA_MESENCHYMAL | 2.67E-24 | 35.5 | 1.10E-20 |
| c2_SCHUETZ_BREAST_CANCER_DUCTAL_INVASIVE_UP | 5.08E-23 | 25.5 | 1.39E-19 |
| c2_PID_INTEGRIN1_PATHWAY | 1.48E-22 | 86.5 | 3.05E-19 |
| c2_NABA_MATRISOME | 4.76E-22 | 17.6 | 7.82E-19 |
| c2_KEGG_ECM_RECEPTOR_INTERACTION | 4.32E-21 | 66.7 | 5.91E-18 |
| c2_NABA_CORE_MATRISOME | 6.05E-17 | 24.5 | 5.52E-14 |
| c2_PICCALUGA_ANGIOIMMUNOBLASTIC_LYMPHOMA_UP | 3.23E-16 | 25.0 | 2.65E-13 |
| c2_ANASTASSIOU_CANCER_MESENCHYMAL_TRANSITION_SIGNATURE | 2.56E-15 | 56.9 | 1.62E-12 |
| c2_CHARAFE_BREAST_CANCER_LUMINAL_VS_MESENCHYMAL_DN | 4.73E-15 | 13.9 | 2.78E-12 |
