# Supplementary material for: Transcriptional changes induced by bevacizumab combination therapy in responding and non-responding recurrent glioblastoma patients
Source: BMC Cancer. 2017 Apr 18;17:278. doi: 10.1186/s12885-017-3251-3 (PMC5395849; doi:10.1186/s12885-017-3251-3)
Supplement: Supplementary file 8 — Ingenuity Pathway Analysis of activated and inhibited canonical pathways (DOCX 18 kb) [file 12885_2017_3251_MOESM8_ESM.docx]

## Table S6. Ingenuity Pathway Analysis of activated and inhibited canonical pathways

| **TOP CANONICAL PATHWAYS** | ***P*-value** | **Ratio** | **Z-score** | **Molecules** |
| --- | --- | --- | --- | --- |
| G Beta Gamma Signaling | 1.13·10^-4^ | 9.9 % 8/81 | 2.1 | ADCY1,PRKCE,GNAO1,GNG3,GNG4,PRKCB, PRKAR1B,PRKCZ |
| Role of NFAT in Cardiac Hypertrophy | 6.52·10^-4^ | 6.3 % 10/158 | 2.8 | ADCY1,PRKCE,SLC8A2,PPP3R1,GNG3,GNG4,PPP3CB,  PRKCB,PRKAR1B,PRKCZ |
| Synaptic Long Term Potentiation | 6.75·10^-4^ | 7.6 % 8/105 | 2.8 | ADCY1,PRKCE,PPP3R1,PPP3CB,PRKCB,PRKAR1B,  PRKCZ,GRIN1 |
| Dopamine-DARPP32 Feedback in cAMP Signaling | 9.26·10^-4^ | 6.6 % 9/137 | 2.1 | ADCY1,PRKCE,PPP3R1,PPP3CB,PRKCB,PRKAR1B, KCNJ10,PRKCZ,GRIN1 |
| Androgen Signaling | 2.11·10^-3^ | 7.1 % 7/98 | 2.0 | GNAO1,GNG3,GNG4,PRKAR1B,PRKCB,PRKCE,PRKCZ |
| CREB Signaling in Neurons | 2.19·10^-3^ | 5.8 % 9/155 | 2.4 | ADCY1,PRKCE,GNAO1,GNG3,GNG4,PRKCB, PRKAR1B,PRKCZ,GRIN1 |
| P2Y Purigenic Receptor Signaling Pathway | 3.65·10^-3^ | 6.5 % 7/108 | 2.2 | ADCY1,PRKCE,GNG3,GNG4,PRKCB,PRKAR1B,PRKCZ |
| Melatonin Signaling | 4.08·10^-3^ | 8.6 % 5/58 | 2.2 | PRKCE,GNAO1,PRKCB,PRKAR1B,PRKCZ |
| Corticotropin Releasing Hormone Signaling | 6.42·10^-3^ | 6.6 % 6/91 | 2.4 | ADCY1,PRKCE,GNAO1,PRKCB,PRKAR1B,PRKCZ |
| IL-3 Signaling | 7.08·10^-3^ | 7.6 % 5/66 | 2.2 | PRKCE,PPP3R1,PPP3CB,PRKCB,PRKCZ |
| Calcium Signaling | 7.40·10^-3^ | 5.7 % 7/123 | 2.2 | ATP2B2,SLC8A2,PPP3R1,PPP3CB,PRKAR1B,ACTA2, GRIN1 |
| GNRH Signaling | 2.20·10^-2^ | 5.0 % 6/119 | 2.4 | ADCY1,PRKCE,PRKCB,PRKAR1B,PRKCZ,DNM3 |
| Neuropathic Pain Signaling In Dorsal Horn Neurons | 2.25·10^-2^ | 5.7 % 5/88 | 2.2 | PRKCE,PRKCB,PRKAR1B,PRKCZ,GRIN1 |
| Renin-Angiotensin Signaling | 3.51·10^-2^ | 5.1 % 5/99 | 2.2 | ADCY1,PRKCE,PRKCB,PRKAR1B,PRKCZ |
| Integrin Signaling | 4.64·10^-2^ | 3.9 % 7/179 | -2.2 | ITGA1,ACTA2,TSPAN7,ARPC1B,PFN2,ITGA5,ITGA7 |
| eNOS Signaling | 4.96·10^-2^ | 4.6 % 5/109 | 2.2 | ADCY1,PRKCE,PRKCB,PRKAR1B,PRKCZ |
