# Supplementary material for: Transcriptional changes induced by bevacizumab combination therapy in responding and non-responding recurrent glioblastoma patients
Source: BMC Cancer. 2017 Apr 18;17:278. doi: 10.1186/s12885-017-3251-3 (PMC5395849; doi:10.1186/s12885-017-3251-3)
Supplement: Supplementary file 9 — Ingenuity Pathway Analysis of activated and inhibited up-stream regulators (DOCX 15 kb) [file 12885_2017_3251_MOESM9_ESM.docx]

## Table S7. Ingenuity Pathway Analysis of activated and inhibited up-stream regulators

| **UPSTREAM REGULATORS** | ***P*-value** | **Z-score** | **Down-stream effector molecules** |
| --- | --- | --- | --- |
| TGFB1 | 8.36E-10 | -4.0 | TIMP1, TGFBI, SERPINH1, SERPINE1, MMP13, ITGA5, ITGA1, IGFBP3, HOOK1, HMOX1, FN1, ENG, COL6A3, COL5A1, COL3A1, COL1A2, COL1A1, CDKN1A, BGN, ACTA2, TSPAN7, THBS1 |
| ERBB2 | 6.75E-03 | -2.4 | ITGA5, FN1, COL6A3, COL5A, COL1A1, CDKN1A, THBS1 |
| ERK | 6.13E-03 | -2.2 | THBS1, SERPINE1, MMP14, MMP13, COL1A1 |
| SMAD3 | 3.88E-06 | -2.2 | MMP13, FN1, COL3A1, COL1A1, COL1A2, COL1A1, CDKN1A, SERPINE1 |
| estrogen receptor | 1.27E-04 | 2,1 | CALB2, COL4A1, COL4A2, COL5A1, FGF12, FN1, LOXL2, MMP14, PCDH7, SERPINE1, TIMP1 |
| SPDEF | 2.92E-05 | 2,6 | COL1A1, COL4A1, COL4A2, COL5A1, COL6A3, ITGA5, SERPINE1 |
