# Supplementary material for: Transcriptional changes induced by bevacizumab combination therapy in responding and non-responding recurrent glioblastoma patients
Source: BMC Cancer. 2017 Apr 18;17:278. doi: 10.1186/s12885-017-3251-3 (PMC5395849; doi:10.1186/s12885-017-3251-3)
Supplement: Supplementary file 10 — Mechanistic network of inhibited TGF-β1 and the three most interconnected regulators (SMAD3, HIF1A and PPARG) of downstream molecules identified differentially expressed in responders at the time of progression (DOCX 122 kb) [file 12885_2017_3251_MOESM10_ESM.docx]

Figure S2. Mechanistic network of inhibited TGF-β1 and the three most interconnected regulators (SMAD3, HIF1A and PPARG) of downstream molecules identified differentially expressed in responders at the time of progression.
