# Supplementary material for: Transcriptional changes induced by bevacizumab combination therapy in responding and non-responding recurrent glioblastoma patients
Source: BMC Cancer. 2017 Apr 18;17:278. doi: 10.1186/s12885-017-3251-3 (PMC5395849; doi:10.1186/s12885-017-3251-3)
Supplement: Supplementary file 11 — Hematoxylin-eosin staining of two representative responding glioblastomas before and after bevacizumab therapy (DOCX 2297 kb) [file 12885_2017_3251_MOESM11_ESM.docx]

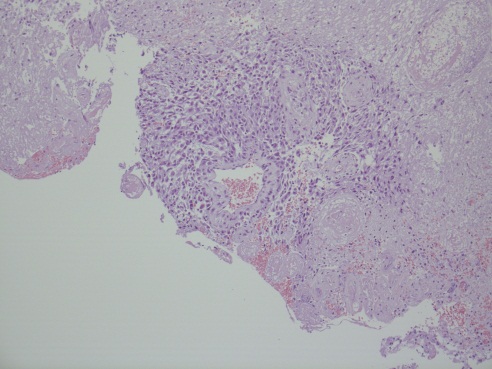

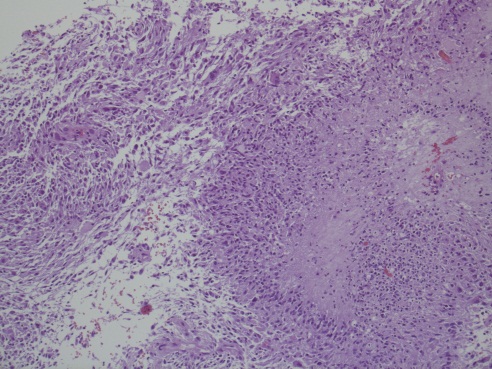

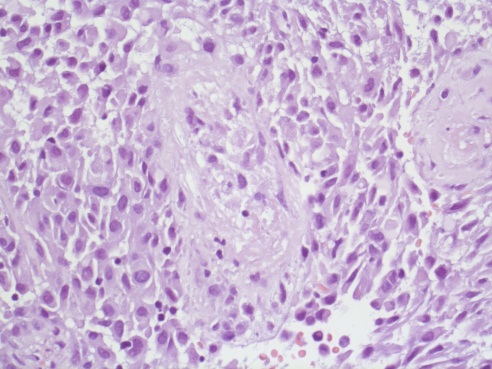

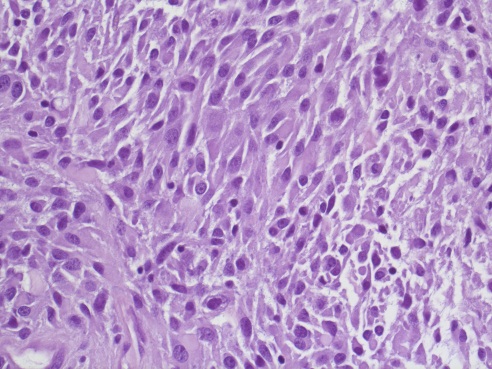

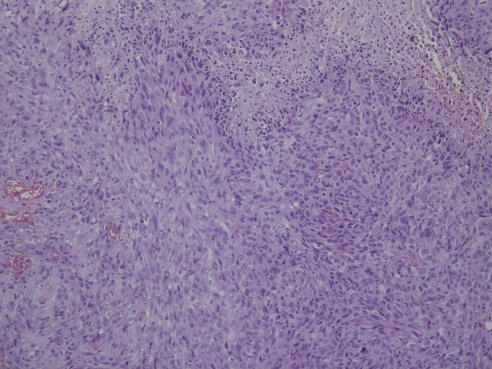

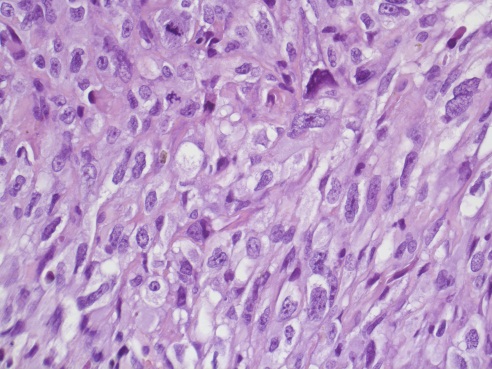

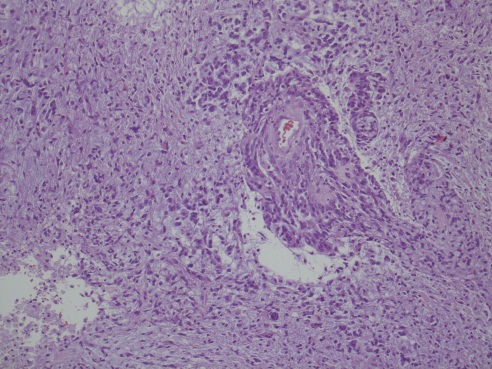

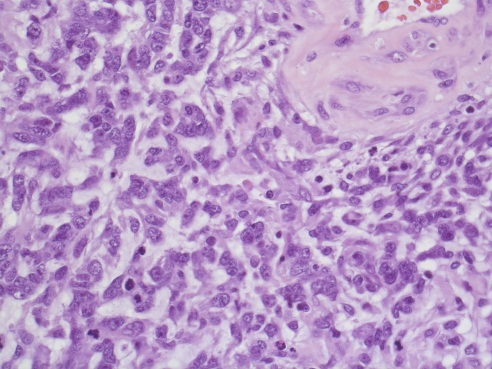


A

C

B

D

F

E

G

H

Before bevacizumab

After bevacizumab

Patient 1

Patient 2

Figure S3. Hematoxylin-eosin staining of two representative responding glioblastomas before and after bevacizumab therapy. Overviews (×100) are shown in A, B, E, F and perivascular areas (×400) of corresponding samples are shown below in C, D, G, H.
